# circSMARCA5 Functions as a Diagnostic and Prognostic Biomarker for Gastric Cancer

**DOI:** 10.1155/2019/2473652

**Published:** 2019-03-06

**Authors:** Juan Cai, Zhiqiang Chen, Xueliang Zuo

**Affiliations:** ^1^Department of Oncology, The First Affiliated Hospital, Yijishan Hospital of Wannan Medical College, Wuhu, Anhui, China; ^2^Hepatobiliary Center, The First Affiliated Hospital of Nanjing Medical University, Key Laboratory of Liver Transplantation, Chinese Academy of Medical Sciences, NHC Key Laboratory of Liver Transplantation, Nanjing, Jiangsu, China; ^3^Department of Gastrointestinal Surgery, The First Affiliated Hospital, Yijishan Hospital of Wannan Medical College, Wuhu, Anhui, China

## Abstract

**Background:**

Circular RNAs have been implicated in various malignancies and can function as potential biomarkers for cancers. Reportedly, circSMARCA5 was downregulated in hepatocellular carcinoma and glioblastoma multiforme, but upregulated in prostate cancer. The functional roles and clinical significance of circSMARCA5 still remain unknown in the context of gastric cancer (GC).

**Methods:**

Expression levels of circSMARCA5 were detected by qRT-PCR. Clinical data including patient basic information, clinicopathological features, and survival data were obtained. The Kaplan-Meier methods, multivariate Cox regression models, and the receiver operating characteristic curve were used to assess the clinical significance of circSMARCA5 in GC. Cell proliferation assays and transwell assays were performed to elucidate the functional roles of circSMARCA5 in GC.

**Results:**

The circSMARCA5 level was decreased in GC tissues and cell lines. The low expression level of circSMARCA5 was correlated to poorer overall survival and disease-free survival. Low circSMARCA5 expression was revealed as an independent unfavorable predictive factor for GC. The results indicated that circSMARCA5 had a moderate ability for discrimination between GC patients and controls with an area under the curve of 0.806. Upregulation of circSMARCA5 dampened the proliferation, migration, and invasion of GC cells, whereas circSMARCA5 knockdown promoted GC progression.

**Discussion:**

Our results demonstrated that circSMARCA5 was decreased and exerted tumor-suppressive effects in GC. circSMARCA5 can function as a potential biomarker for GC prognosis and diagnosis.

## 1. Introduction

Gastric cancer (GC) is one of the most commonly diagnosed malignancies and a leading cause of death worldwide. The annual new cases of GC were 26,240, and the estimated deaths were 10,800 persons in the United States [1]. Despite currently available first-line treatments, the five-year survival rate of GC remains dismal. Early diagnosis and systematic treatment significantly increase the survival outcomes of GC patients. Gastroscopy provides the highest diagnostic accuracy; however, its invasiveness and inconvenience limit its use for preliminary diagnosis [2]. Though widely used, carcinoembryonic antigen (CEA) exhibits low sensitivity and specificity particularly in the early stages of GC [3]. Therefore, improvement in GC prevention and early detection is of great significance.

Circular RNAs (circRNAs) are a diverse class of RNA transcripts with no apparent protein-coding role [4]. circRNAs are engaged in numerous biological processes across every branch of life [5]. Specific patterns of circRNA expression coordinate cell differentiation, development, and disease [6–8]. It has been widely recognized that circRNAs play crucial roles in tumorigenesis and cancer progression [9]. Several circRNAs have been reported as biomarkers for GC prognosis and diagnosis [10–13]. circSMARCA5 originates from exons 15 and 16 of the *SMARCA5* gene which encodes the specific chromatin-remodeling protein SNF2H and modulates the chromatin structure for DNA transcription and repair [14]. circSMARCA5 has been implicated to be downregulated in hepatocellular carcinoma and glioblastoma multiforme, but increased in prostate cancer [15–17]. However, the involvement of circSMARCA5 in GC has not yet been documented, which prompted us to explore the role of circSMARCA5 and its clinical value in GC.

In the current study, we aimed to investigate the expression level of circSMARCA5 in GC tissues and cell lines, as well as its predictive value for the prognosis and diagnosis of GC patients. Our results revealed that circSMARCA5 was downregulated in GC tissues compared with the corresponding nontumor tissues. The circSMARCA5 expression level was confirmed to be an independent prognostic factor for overall survival (OS) and disease-free survival (DFS) in GC patients. Moreover, the diagnostic value of circSMARCA5 for GC was observed in the present study. Functional experiments suggested that circSMARCA5 could function as a tumor suppressor and contribute to the development of circRNA-directed diagnostics and therapeutics of GC.

## 2. Materials and Methods

### 2.1. Patient Samples

A total of 60 GC tissues and their paired adjuvant nontumor mucosa were obtained from patients who underwent radical surgery at the Department of Gastrointestinal Surgery, the First Affiliated Hospital of Wannan Medical College. All tissue samples were stored in liquid nitrogen until RNA extraction. Peripheral blood samples were collected from preoperative GC patients (*n* = 50) and healthy controls (*n* = 50). Blood was collected in ethylenediaminetetraacetic acid anticoagulation tubes and processed for plasma within 2 h using standard procedures [18]. The tube was inverted three times and spun at 3,500 rpm for 8 min and then banked at -80°C until use. All GC patients were histopathologically confirmed by at least two pathologists. The postoperative pathological stages were determined according to the eighth edition of the cancer staging criteria of the American Joint Committee on Cancer (AJCC). None of the patients had received preoperative therapy. Written informed consents were acquired from all patients before operation. This study was in accordance with the ethical guidelines of the 1975 Declaration of Helsinki and approved by the ethical review board of the First Affiliated Hospital of Wannan Medical College.

### 2.2. Cell Lines and Cell Culture

The normal human gastric mucosal epithelial cell line (GES-1) and six GC cell lines (MGC803, MKN45, MKN74, AGS, BGC823, and SGC7901) were acquired from the Shanghai Institute of Biochemistry and Cell Biology, Chinese Academy of Sciences (Shanghai, China). Cells were cultured in RPMI-1640 (Gibco, Carlsbad, CA, USA) supplemented with 10% fetal bovine serum (Invitrogen, Carlsbad, CA, USA), 100 U/mL penicillin, and 100 *μ*g/mL streptomycin (Invitrogen). Cells were maintained in a humidified atmosphere at 37°C with 5% CO_2_.

### 2.3. RNA Preparation and RNase R Treatment

Total RNA from tissues and cells was extracted using a TRIzol reagent (Invitrogen). RNase R (Epicentre Technologies, Madison, WI, USA) was employed to degrade linear RNAs. Briefly, 2 *μ*g of total RNA was mixed with 3 U/*μ*g of RNase R. The samples were then incubated at 37°C for 15 min.

### 2.4. Quantitative Real-Time Polymerase Chain Reaction (qRT-PCR)

Total RNA was reverse transcribed into cDNA using the PrimeScript RT Master Mix (TaKaRa, Dalian, China) according to the manufacturer's protocols. qRT-PCR was performed using the TB Green Premix Ex Taq (TaKaRa) on an ABI 7900HT (Applied Biosystems, Foster City, CA, USA). GADPH served as an internal control for circRNA and mRNA. The primer sequences used for qRT-PCR were listed as follows: circSMARCA5, 5′-CTCCAAGATGGGCGAAAG-3′ (forward), 5′-TGTGTTGCTCCATGTCTAATCA-3′ (reverse); SMARCA5, 5′-TGCAAACTGACCGGGCAAATA-3′ (forward), 5′-TCGCCAACGGATAGTAAGTTCT-3′ (reverse); and GAPDH, 5′-CAGGAGGCATTGCTGATGAT-3′ (forward), 5′-GAAGGCTGGGGCTCATTT-3′ (reverse).

### 2.5. Establishment of Stably circSMARCA5-Overexpressed and circSMARCA5-Silenced Cell Lines

We purchased the lentiviruses overexpressing circSMARCA5 (LV-circSMARCA5), lentiviruses with circSMARCA5 knockdown (sh-circSMARCA5), and corresponding negative controls from GeneChem (Shanghai, China). For circSMARCA5 overexpression, SGC7901 or BGC823 cell lines were infected with LV-circSMARCA5 or LV-NC lentiviruses with polybrene (5 mg/mL). For circSMARCA5 knockdown, we infected MGC803 and MKN45 cells with sh-circSMARCA5 and sh-NC lentiviruses in the presence of 5 mg/mL of polybrene. The sequence of sh-circSMARCA5 was as follows: 5′-AAACAAAAGGGAGGCTTGTTT-3′. Sable cell lines were selected using puromycin (5 *μ*g/mL) for one week.

### 2.6. Cell Counting Kit-8 (CCK-8) Assays

Cell proliferation was examined using CCK-8 (Dojindo Laboratories, Kumamoto, Japan) in accordance with the manufacturer's instructions. In brief, a total of 100 *μ*L of RPMI-1640 culture medium containing 1 × 10^3^ GC cells was seeded onto 96-well plates. At the indicated time (1 d, 2 d, 3 d, 4 d, and 5 d), 10 *μ*L of CCK-8 solution was added into each well. After incubation in the dark at 37°C for 2 h, absorbance at 450 nm was detected and recorded. The experiments were repeated three times.

### 2.7. Transwell Assays

To assess the effect of circSMARCA5 on GC cell migration and invasion, we performed transwell assays. For migration assays, 250 *μ*L of serum-free RPMI-1640 containing 2 × 10^4^ GC cells was added in the upper chamber of the transwell inserts. For cell invasion, the same amount of cells was seeded into the upper chamber coated with a mixture of 50 *μ*L of Matrigel (BD Biosciences, San Jose, CA, USA) and 50 *μ*L of RPMI-1640. A total of 500 *μ*L of RPMI-1640 containing 10% FBS was added in the lower chamber. After incubation for one day, the cells in the upper chamber were removed by cotton tips. The migrated or invaded cells were then fixed, stained, and photographed. The experiments were performed in triplicate.

### 2.8. Statistical Analysis

All statistical analyses were performed using the SPSS 24.0 software (IBM Corp., Armonk, NY, USA) and GraphPad Prism software (GraphPad Software, La Jolla, CA, USA). Continuous data were shown as the means ± standard errors of the mean, and differences between groups were analyzed by two-sided Student's *t*-test. For categorical data, the Fisher exact test and chi-square test were employed. The area under the receiver operating characteristic (ROC) curve (AUC) was used to evaluate the diagnostic value of circSMARCA5. The Kaplan-Meier method with a log-rank test was performed to assess OS and DFS, and the Cox proportional hazard model was used for multivariate analysis. *P* values < 0.05 were considered statistically significant.

## 3. Results

### 3.1. circSMARCA5 Expression Level Is Downregulated in GC Tissues and Cell Lines

**Figure 1:**
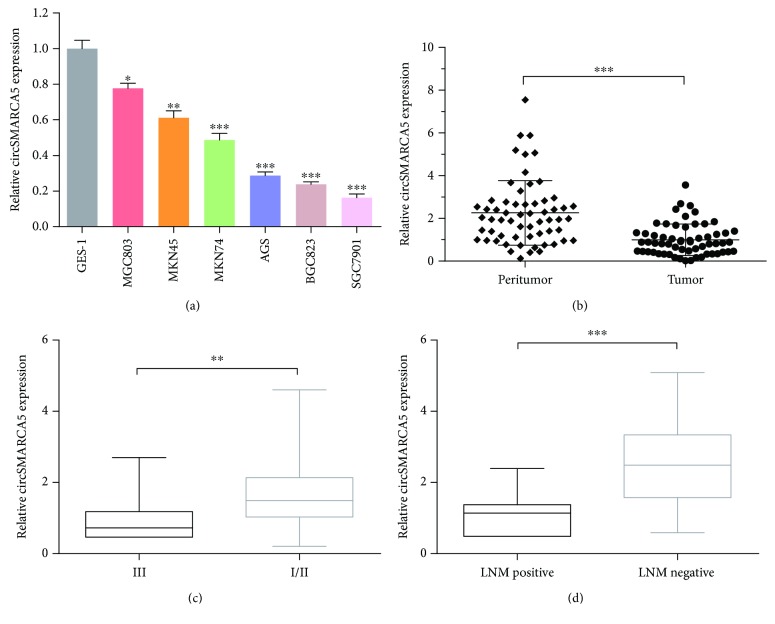
Downregulation of circSMARCA5 in gastric cancer tissues and cell lines. (a) circSMARCA5 expression levels in six GC cell lines (MGC803, MKN45, MKN74, AGS, BGC823, and SGC7901) and normal gastric epithelial cell line (GES-1) were assessed by qRT-PCR. (b) circSMARCA5 expression levels in 60 pairs of gastric cancer tissues compared with matched adjacent noncancerous tissues. (c) The expression levels of circSMARCA5 in the AJCC stage III group and I/II group. (d) The expression levels of circSMARCA5 in the lymph node metastasis- (LNM-) positive group and LNM-negative group. Data are presented as mean ± S.E.M.; ^∗^*P* < 0.05, ^∗∗^*P* < 0.01, and ^∗∗∗^*P* < 0.001.

We first examined the expression of circSMARCA5 in six GC cell lines (MGC803, MKN45, MKN74, AGS, BGC823, and SGC7901) and the normal gastric epithelial cell line (GES-1). Compared with GES-1, the expression levels of circSMARCA5 in all six GC cell lines were significantly decreased (Figure 1(a)). The expression level of circSMARCA5 was detected in 60 matched GC tissues and adjacent mucosa. Results of qRT-PCR indicated that circSMARCA5 was aberrantly decreased in GC tissues when compared to paired adjacent mucosa (*P* < 0.001, Figure 1(b)). After dividing 60 patients into two groups based on the AJCC stage, the decreased expression level of circSMARCA5 was observed in the AJCC stage III group compared to the AJCC stage I/II group (*P* < 0.01, Figure 1(c)). We next separated the patients according to the status of lymph node metastasis (LNM) and found a lower level of circSMARCA5 in the LNM-positive group than that in the LNM-negative group (*P* < 0.001, Figure 1(d)). Taken together, the results herein revealed that the expression of circSMARCA5 was downregulated in GC tissues and cell lines.

### 3.2. Correlation of circSMARCA5 Expression with Clinicopathological Features

Based on the median value of circSMARCA5 expression in GC tissues, we categorized the enrolled patients into the high-expression group (*n* = 30) and low-expression group (*n* = 30). As shown in Table 1, we found that circSMARCA5 expression was significantly associated with differentiation (*P* = 0.019), LNM (*P* = 0.015), vascular invasion (*P* = 0.009), and AJCC stage (*P* = 0.017). Other clinicopathological variables including age (*P* = 0.589), gender (*P* = 0.382), tumor location (*P* = 0.785), and tumor size (*P* = 0.267), however, were not correlated to the expression level of circSMARCA5.

### 3.3. The Prognostic Value of circSMARCA5 for GC Patients

**Figure 2:**
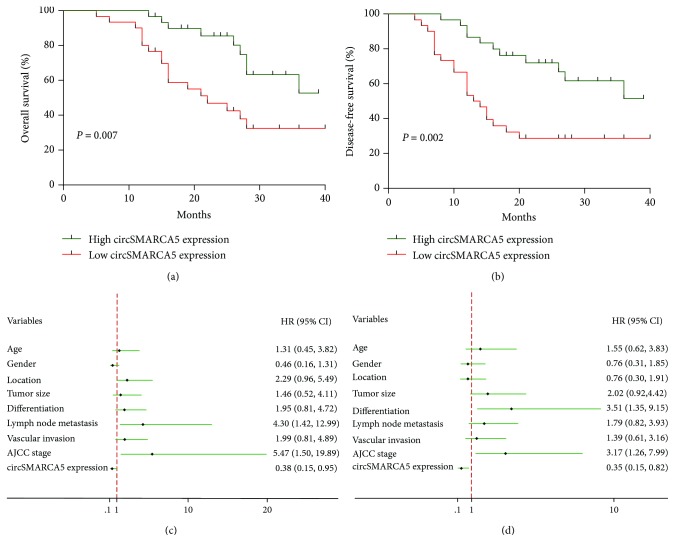
The prognostic value of circSMARCA5 in GC patients. (a) The Kaplan-Meier curves for overall survival. (b) The Kaplan-Meier curves for disease-free survival. (c) Cox proportional hazard regression analysis showing the independent risk factors for overall survival. (d) Cox proportional hazard regression analysis showing the independent risk factors for disease-free survival.

We further evaluated the impact of circSMARCA5 on the survival outcomes of GC patients. When the included GC patients were separated into two groups according to the median value of circSMARCA5 expression levels, substantial differences were observed in patient prognosis between the low circSMARCA5 expression group and the high circSMARCA5 expression group. The Kaplan-Meier survival analysis revealed that the low expression level of circSMARCA5 was associated with unfavorable OS and DFS (OS: *P* = 0.007, DFS: *P* = 0.002; Figures 2(a) and 2(b)). As shown in Table 2, univariate analysis identified four prognostic factors for OS: LNM (*P* = 0.011), vascular invasion (*P* = 0.016), AJCC stage (*P* < 0.001), and circSMARCA5 expression (*P* = 0.007). A total of 5 variables were revealed as prognostic factors for DFS: differentiation (*P* = 0.026), LNM (*P* = 0.007), vascular invasion (*P* = 0.030), AJCC stage (*P* = 0.002), and circSMARCA5 expression (*P* = 0.002). Several other clinicopathological features, such as age (OS: *P* = 0.930; DFS: *P* = 0.892), gender (OS: *P* = 0.794; DFS: *P* = 0.809), tumor location (OS: *P* = 0.915; DFS: *P* = 0.381), tumor size (OS: *P* = 0.144; DFS: *P* = 0.120), and differentiation (OS: *P* = 0.056), were not statistically significant according to the results of univariate analysis. To further determine the independent predictive factors for OS and DFS, we performed the multivariate Cox regression analysis. LNM [hazard ratio (HR) 4.296, 95% confidence interval (CI) 1.420-12.993, *P* = 0.010], AJCC stage (HR 5.465, 95% CI 1.501-19.892, *P* = 0.010), and circSMARCA5 expression (HR 0.383, 95% CI 0.154-0.954, *P* = 0.039) were found to be independent prognostic factors for OS by multivariate analysis (Figure 2(c) and Table 2). Three clinicopathological characteristics were verified as independent predictive factors for DFS: differentiation (HR 3.514, 95% CI 1.349-9.153, *P* = 0.010), AJCC stage (HR 3.175, 95% CI 1.261-7.995, *P* = 0.014), and circSMARCA5 expression (HR 0.347, 95% CI 0.146-0.823, *P* = 0.016) (Figure 2(d) and Table 2). Collectively, low circSMARCA5 expression was associated with poor OS and DFS in GC patients, and the expression level of circSMARCA5 was found to be an independent prognostic factor for the survival of patients diagnosed with GC.

### 3.4. The Diagnostic Accuracy of circSMARCA5 for GC Patients

**Figure 3:**
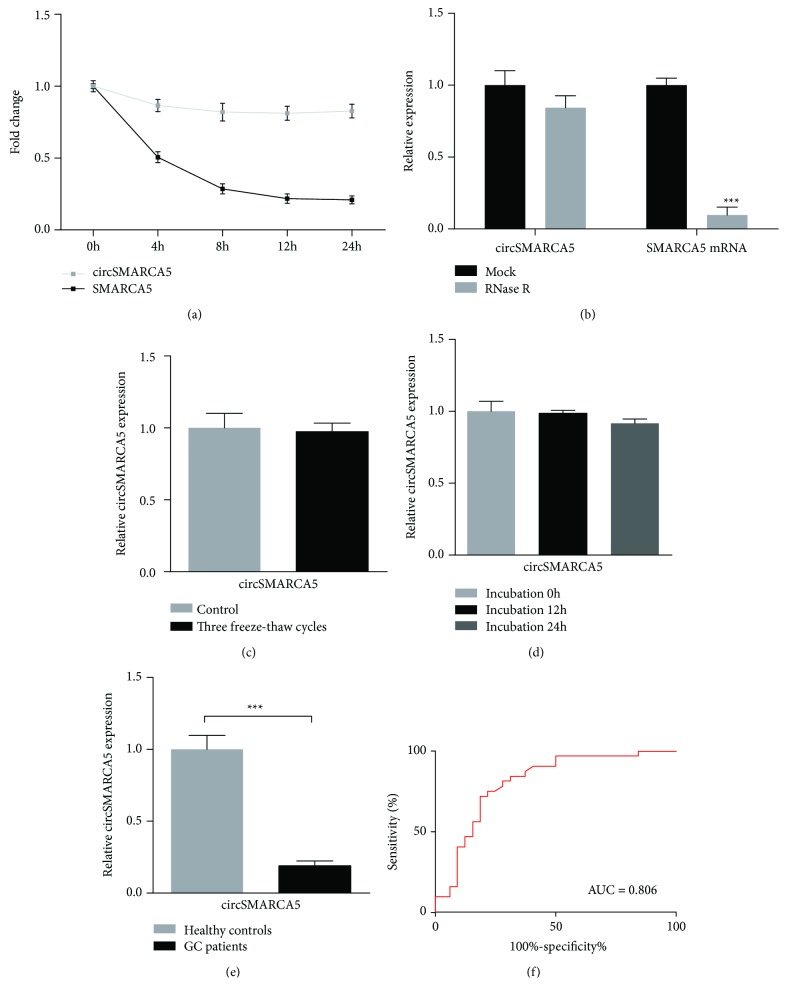
The stability and diagnostic accuracy of circSMARCA5 for GC. (a) The qRT-PCR assay was used to show the abundance of circSMARCA5 and SMARCA5 mRNAs in SGC7901 treated with actinomycin D. (b) circSMARCA5 and SMARCA5 mRNA expression levels were examined by qRT-PCR after treatment with RNase R in SGC7901. (c) No statistically significant difference was observed in circSMARCA5 expression between plasma samples treated with three freeze-thaw cycles and the control group. (d) There was no difference of circSMARCA5 expression when plasma was incubated at room temperature for 0, 12, and 24 h. (e) The expression level of plasma circSMARCA5 was lower in GC patients than in healthy controls. (f) The area under the ROC curve (AUC) was 0.806. Data are shown as mean ± S.E.M.; ^∗∗∗^*P* < 0.001.

We first examined the stability of circSMARCA5 using the transcription inhibitor actinomycin D. The results showed that the half-life of circSMARCA5 exceeded 24 h and the half-life of linear SMARCA5 was approximately 4 h (Figure 3(a)). After RNase R treatment, the abundance of circSMARCA5 and linear SMARCA5 was detected using qRT-PCR. The data indicated that circSMARCA5 was resistant to RNase R treatment, further confirming the circular structure of circSMARCA5 (Figure 3(b)). As shown in Figures 3(c) and 3(d), plasma circSMARCA5 remained stable in harsh conditions, including repeated freeze-thaw cycles and incubation at room temperature. To assess the diagnostic accuracy of circSMARCA5 in patients with GC, we detected the plasma expression level of circSMARCA5 in 50 GC patients and 50 healthy controls. Consistent with the expression patterns in tissue samples, the decreased expression level of plasma circSMARCA5 was observed in GC patients compared with healthy controls (Figure 3(e)). ROC curve analysis was used to evaluate the diagnostic value of circSMARCA5 for GC. The results indicated that circSMARCA5 had a moderate ability for discrimination between GC patients and controls with an AUC of 0.806 (Figure 3(f)).

### 3.5. circSMARCA5 Inhibits the GC Cell Proliferation, Migration, and Invasion

**Figure 4:**
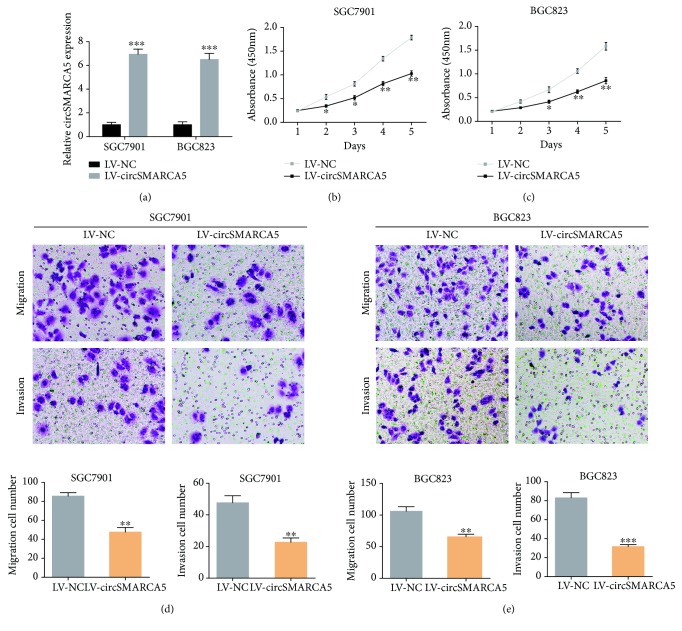
circSMARCA5 overexpression inhibits cell proliferation, migration, and invasion in GC. (a) Lentiviruses were used to upregulate the expression of circSMARCA5 in SGC7901 and BGC823 cells. (b) CCK-8 assays were performed to examine the effects of circSMARCA5 on SGC7901 cell proliferation. (c) CCK-8 assays were performed to examine the effects of circSMARCA5 on BGC823 cell proliferation. (d) Transwell assays were conducted to examine the effects of circSMARCA5 on SGC7901 cell migration and invasion. (e) Transwell assays were conducted to examine the effects of circSMARCA5 on BGC823 cell migration and invasion.

To explore the functional roles of circSMARCA5, we overexpressed circSMARCA5 in two circSMARCA5 low-expressing cell lines (SGC7901 and BGC823). As indicated in Figure 4(a), the efficiency of circSMARCA5 overexpression lentiviruses was detected by qRT-PCR. We then performed CCK-8 assays to examine the proliferative capacity of LV-circSMARCA5 cells. The results showed that circSMARCA5 overexpression significantly damped the cell viability in SGC7901 and BGC823 cells (Figures 4(b) and 4(c)). Moreover, transwell assays were carried out to assess the migration and invasion in cells overexpressing circSMARCA5. As indicated in Figures 4(d) and 4(e), circSMARCA5 overexpression led to fewer migrated and invaded GC cells.

**Figure 5:**
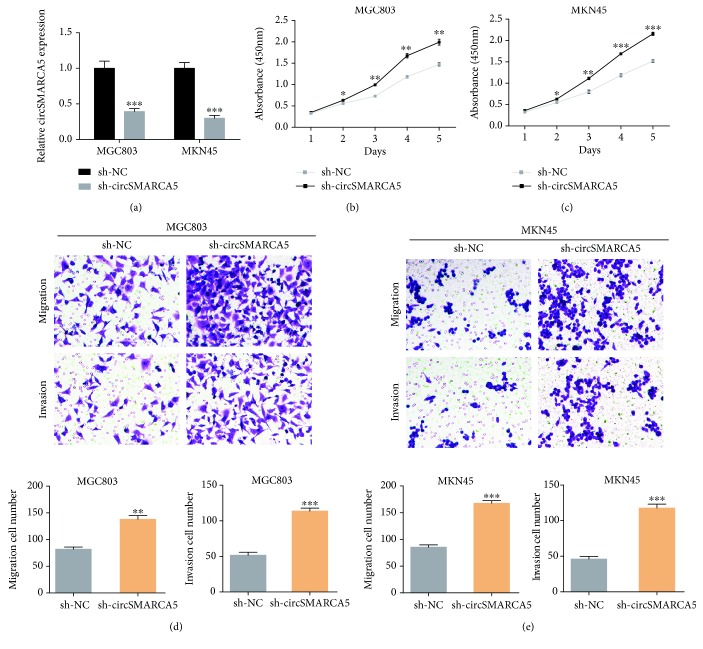
circSMARCA5 knockdown promotes GC proliferation, migration, and invasion. (a) The knockdown efficiency of circSMARCA5 was determined using qRT-PCR in MGC803 and MKN45 cells. (b) CCK-8 assays were conducted to assess the effects of circSMARCA5 on MGC803 cell proliferation. (c) CCK-8 assays were conducted to assess the effects of circSMARCA5 on MKN45 cell proliferation. (d) Transwell assays were performed to investigate the effects of circSMARCA5 on MGC803 cell migration and invasion. (e) Transwell assays were performed to investigate the effects of circSMARCA5 on MKN45 cell migration and invasion.

In addition, we knocked down the expression level of circSMARCA5 in MGC803 and MKN45 cells using lentivirus (Figure 5(a)). CCK-8 and transwell assays were performed to assess the effects of circSMARCA5 on GC progression. As shown in Figures 5(b) and 5(c), MGC803 and MKN45 cells transfected with sh-circSMARCA5 presented higher proliferative capacity when compared to the controls. Elevated migration and invasion ability was observed in GC cells with circSMARCA5 knockdown as compared to the control cells (Figures 5(d) and 5(e)). Taken together, we performed functional experiments in GC cells with circSMARCA5 overexpression or knockdown, and the results demonstrated that circSMARCA5 exerted a tumor-suppressive effect on GC.

## 4. Discussion

The number of circRNAs found to be differentially expressed in malignancies is continuously increasing. Emerging evidence has indicated that circRNAs play an indispensable role in the carcinogenesis, progression, and clinical outcomes of various human cancers [19–21]. As reported, circEPSTI1 promotes triple-negative breast cancer cell proliferation and apoptosis through sponging miR-4753 and miR-6809 to regulate the BCL11A level [22]. In glioblastoma, circNT5E serves as a sponge against miR-422a to modulate cell proliferation, migration, and invasion [23]. circLARP4 suppresses the growth and metastasis of GC via binding to miR-424-5p and regulating the Hippo signaling pathway [24]. Given the crucial roles that circRNAs play in cancers, the diagnostic and prognostic value of circRNAs is among one of the research hotspots.

circRNAs have been reported to serve as potential diagnostic and prognostic biomarkers for human cancers due to their stability [25–27]. With regard to GC, hsa_circ_002059 is decreased and functions as a potential marker for GC prognosis [12]. Upregulated circPVT1 has been suggested as a potential independent predictor for OS and DFS in patients diagnosed with GC [28]. A lower level of hsa_circ_0000190 is observed in the plasma specimen from GC patients and is associated with tumor size, lymphatic metastasis, and distal metastasis. The diagnostic accuracy of hsa_circ_0000190 is superior to carbohydrate antigen 19-9 (CA19-9) and CEA [29]. In addition, multiple circRNAs including hsa_circ_100269, hsa_circ_104916, hsa_circ_101308, hsa_circ_0001017, and hsa_circ_0001895 have been proposed as potential candidates for GC diagnosis and prognosis [30–32].

In the current study, we found that the expression level of circSMARCA5 was markedly downregulated in GC tissues compared to adjacent nontumor tissues. Similarly, the circSMARCA5 expression was lower in GC cell lines. Furthermore, the circSMARCA5 expression level was significantly correlated to differentiation, LNM, vascular invasion, and AJCC stage. We then adopted the Kaplan-Meier methods to assess the impact of circSMARCA5 on patient survival. Compared with GC patients with a high circSMARCA5 expression level, patients with a low circSMARCA5 expression had shorter OS and DFS. Multivariate survival analyses showed that LNM, AJCC stage, and circSMARCA5 expression were independent predictive factors for OS in GC patients. With regard to DFS, circSMARCA5 expression was also verified as an independent prognostic factor in GC.

Recently, several studies indicated the crucial role of circSMARCA5 in the tumorigenesis of hepatocellular carcinoma, glioblastoma multiforme, and prostate cancer. However, the expression pattern and functional roles of circSMARCA5 in GC remain an enigma. circSMARCA5 is predominantly cytoplasmic enriched. *In vitro* and *in vivo* experiments show that circSMARCA5 suppresses hepatocellular carcinoma proliferation and metastasis by sponging miR-17-3p and miR-181b-5p, subsequently promoting the expression of tumor suppressor TIMP3 [15]. Overexpressing circSMARCA5 in glioblastoma multiforme cells significantly decreases their migration via regulating the SRSF1/SRSF3/PTB axis [16]. In prostate cancer, circSMARCA5 functions as an oncogenic circRNA through suppression of apoptosis and promotion of the cell cycle [17]. Given that circSMARCA5 is a crucial regulator in cancer growth and metastasis, we postulated that circSMARCA5 might play an important role in GC progression. Using gain-of-function and loss-of-function experiments, we found that circSMARCA5 exerted tumor-suppressive activities against GC.

To evaluate the potential of circSMARCA5 for GC diagnosis, we investigated the stability of circSMARCA5 in GC cells and plasma samples. Our data showed that circSMARCA5 was highly stable and can act as a potential biomarker in GC patients. Moreover, we detected the plasma level of circSMARCA5 in GC patients and healthy controls. The results showed a potential value of circSMARCA5 for the diagnosis of GC patients, with an AUC of 0.806. The AUC of ROC combines the strengths of sensitivity and specificity and expresses the diagnostic performance as a single term. As previously reported, an AUC with a value ranging between 0.93 and 0.96 is recognized to be excellent and a value from 0.75 to 0.92 is acceptable [33, 34]. CEA and CA19-9 are the most commonly used biomarkers for GC. Although widely used, they are not sufficient because of their deficient sensitivity and specificity [35]. As reported in Wu et al.'s study, the AUC of CEA is 0.671 for GC diagnosis [36]. In addition, the AUC of CA19-9 is 0.563 for GC as previously reported [37]. With an AUC of 0.806, circSMARCA5 may be a promising biomarker for diagnosing potential patients with GC. Moreover, it might be of greater diagnostic value if circSMARCA5 is employed in combination with other biomarkers such as CEA and CA19-9.

Despite our endeavors, there were still several limitations in the present study. First, the sample size of the included patients is relatively small, and the enrolled subjects were from a single center in China, which decreased the applicability of the results across different populations. Second, we performed this population-based study to assess the expression patterns of circSMARCA5 and its clinical significance in GC. Our results suggested that circSMARCA5 might possess a tumor-suppressive effect during GC progression. To further confirm our results, *in vivo* animal studies using CRISPR/Cas9 knockout techniques to investigate the functions and underlying molecular mechanisms are necessary in the future. Third, it may be a more diagnostically valid method for GC detection and monitoring if circSMARCA5 is employed in combination with other protein-coding biomarkers and microRNA signatures in GC.

To conclude, the present study revealed that the expression of circSMARCA5 was downregulated in GC and the low circSMARCA5 level was associated with tumor differentiation, LNM, vascular invasion, and AJCC stage. Survival analyses showed that lower circSMARCA5 predicted poorer OS and DFS for GC patients. Based on the results presented herein, circSMARCA5 may serve as a candidate biomarker for GC prognosis and diagnosis. Further investigation into the molecular mechanisms underlying the dysregulation of circSMARCA5 in GC patients is duly warranted.

## Figures and Tables

**Table 1:** Correlation between the circSMARCA5 expression and clinicopathological features of gastric cancer patients (*n* = 60). The median expression level of the circSMARCA5 expression was used as the cutoff value. Data were analyzed by the Fisher exact test or chi-square test.

Clinicopathological features	Low circSMARCA5 expression (*n* = 30)	High circSMARCA5 expression (*n* = 30)	*P*
Age			0.589
<60 years	12	9	
≥60 years	18	21	
Gender			0.382
Female	6	10	
Male	24	20	
Tumor location			0.785
Down	19	21	
Upper/middle	11	9	
Tumor size			0.267
<5 cm	18	23	
≥5 cm	12	7	
Differentiation			0.019^∗^
Well/moderate	9	19	
Poor	21	11	
Lymph node metastasis			0.015^∗^
Negative	6	16	
Positive	24	14	
Vascular invasion			0.009^∗^
No	9	20	
Yes	21	10	
AJCC stage			0.017^∗^
I/II	7	17	
III	23	13	

∗ indicates statistical significance. AJCC: American Joint Committee on Cancer.

**Table 2:** Univariate and multivariable analyses of overall survival and disease-free survival after surgery.

Variables	Overall survival					Disease-free survival				
	Univariate		Multivariate			Univariate		Multivariate		
	Log-rank	*P*	HR	95% CI	*P*	Log-rank	*P*	HR	95% CI	*P*
Age (≥60 years vs. <60 years)	0.008	0.930	1.307	0.447-3.821	0.624	0.019	0.892	1.546	0.624-3.834	0.347
Gender (male vs. female)	0.068	0.794	0.460	0.162-1.307	0.145	0.058	0.809	0.763	0.314-1.851	0.549
Tumor location (upper/middle vs. down)	0.011	0.915	2.294	0.958-5.489	0.062	0.769	0.381	0.759	0.302-1.912	0.559
Tumor size (≥5 cm vs. <5 cm)	2.140	0.144	1.457	0.517-4.107	0.477	2.415	0.120	2.015	0.918-4.423	0.081
Differentiation (poor vs. well/moderate)	3.658	0.056	1.951	0.807-4.718	0.138	4.953	0.026^∗^	3.514	1.349-9.153	0.010^∗^
Lymph node metastasis (positive vs. negative)	6.420	0.011^∗^	4.296	1.420-12.993	0.010^∗^	7.387	0.007^∗^	1.794	0.818-3.930	0.144
Vascular invasion (yes vs. no)	5.826	0.016^∗^	1.986	0.806-4.893	0.136	4.713	0.030^∗^	1.393	0.613-3.165	0.428
AJCC stage (III vs. I/II)	12.441	< 0.001^∗^	5.465	1.501-19.892	0.010^∗^	9.254	0.002^∗^	3.175	1.261-7.995	0.014^∗^
circSMARCA5 expression (high vs. low)	7.264	0.007^∗^	0.383	0.154-0.954	0.039^∗^	9.440	0.002^∗^	0.347	0.146-0.823	0.016^∗^

∗ indicates statistical significance. HR: hazard ratio; CI: confidence interval; AJCC: American Joint Committee on Cancer.

## Data Availability

The datasets used/or analyzed during the current study are available from the corresponding author on reasonable request.
